# Human Mobility Monitoring in Very Low Resolution Visual Sensor Network

**DOI:** 10.3390/s141120800

**Published:** 2014-11-04

**Authors:** Nyan Bo Bo, Francis Deboeverie, Mohamed Eldib, Junzhi Guan, Xingzhe Xie, Jorge Niño, Dirk Van Haerenborgh, Maarten Slembrouck, Samuel Van de Velde, Heidi Steendam, Peter Veelaert, Richard Kleihorst, Hamid Aghajan, Wilfried Philips

**Affiliations:** 1 Image Processing and Interpretation, Gent University/iMinds, Gent 9000, Belgium; E-Mails: francis.deboeverie@ugent.be (F.D.); meldib@telin.ugent.be (M.E.); jguan@telin.ugent.be (J.G.); xxie@telin.ugent.be (X.X.); jorge.nino@telin.ugent.be (J.N.); dirk.vanhaerenborgh@telin.ugent.be (D.V.H.); mslembro@telin.ugent.be (M.S.); peter.veelaert@ugent.be (P.V.); richard@kleihorst.com (R.K.); Hamid.Aghajan@UGent.be (H.A.); philips@telin.UGent.be (W.P.); 2 Digital Communications, Gent University/iMinds, Gent 9000, Belgium; E-Mails: Samuel.VandeVelde@telin.ugent.be (S.V.V.); Heidi.Steendam@telin.ugent.be (H.S.); 3 Ambient Intelligence Research Lab, David Packard Building, Stanford, CA 94305, USA

**Keywords:** visual sensor network, low resolution imagery, distributed processing, tracking, mobility analysis

## Abstract

This paper proposes an automated system for monitoring mobility patterns using a network of very low resolution visual sensors (30 × 30 pixels). The use of very low resolution sensors reduces privacy concern, cost, computation requirement and power consumption. The core of our proposed system is a robust people tracker that uses low resolution videos provided by the visual sensor network. The distributed processing architecture of our tracking system allows all image processing tasks to be done on the digital signal controller in each visual sensor. In this paper, we experimentally show that reliable tracking of people is possible using very low resolution imagery. We also compare the performance of our tracker against a state-of-the-art tracking method and show that our method outperforms. Moreover, the mobility statistics of tracks such as total distance traveled and average speed derived from trajectories are compared with those derived from ground truth given by Ultra-Wide Band sensors. The results of this comparison show that the trajectories from our system are accurate enough to obtain useful mobility statistics.

## 1. Introduction

The aging society faces tough economic challenges, many of which are related to high labor costs. Population aging induces a growing incidence of cognitive disorders, such as Alzheimer's disease, impaired mobility, *etc*. More and more people require access to 24 h assistance and are therefore sent to care facilities for this reason, at a high economic cost. Often patients are institutionalized earlier than truly necessary because family and caregivers cannot risk the occurrence of serious problems in the absence of caregivers.

This paper focuses on the application of monitoring the condition of patients with slowly evolving medical conditions. These patients are often quite capable to live at home, and caregivers can properly monitor their condition with minimal support. For instance, in patients with Alzheimer's disease, increased wandering behavior may indicate a progression of the disease. Conversely, a decrease in wandering behavior may indicate that the current medication is working. Unfortunately, patients suffering from Alzheimer's disease cannot provide reliable information on this issue, and family members often fail to notice the subtle and gradual changes. As another example, people suffering from poor mobility after hip surgery will not always accurately report changes in mobility patterns. They may be afraid to report a deterioration, which might force them to enter a care facility, or the changes maybe too gradual to notice over a short period.

In this context, an automated system for detecting and measuring mobility patterns would serve the needs of both Alzheimer's disease patients and patients suffering from poor mobility. In Alzheimer's disease patients it can detect wandering behavior. In patients with poor mobility, it can detect changes in speed as well as a reduction or increase in the amount of walking. In fact, such a system can provide health recommendations for healthy but health-conscious people as well. For instance, it may monitor office workers and advise them to take breaks more often if they spend too much time behind the computer.

The system proposed in this paper is based on algorithms developed in a fundamental research project “Multi-camera human behavior monitoring and unusual event detection,” and is currently being developed as part of the project “Little Sister: Low-cost monitoring for care and retail” [1], which focuses on monitoring mobility. It is also one of the core components of the Ambient Assisted Living Joint Programme project “SONOPA: Social Networks for Older adults to Promote an Active life” [2]. In Sonopa, the aim is to derive models for the wellness of the user along four dimensions: social, eating, leisure habits and mobility. Based on these models, health and social activity recommendations are generated and provided through social networks. The models are derived from the analysis of mobility and activity patterns, as extracted from not only our system but also other inputs such as online social networks.

**Figure 1.**
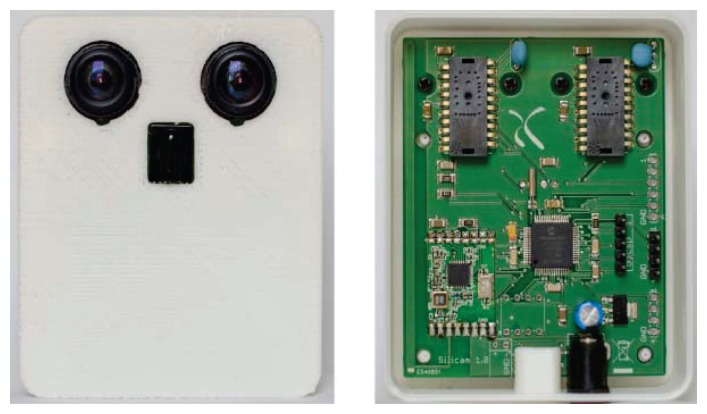
A low resolution visual sensor containing two mouse sensors controlled by a digital signal controller. Each mouse sensor captures an image with a resolution of 30 × 30 pixels.

The first step in measuring and assessing mobility patterns is to acquire mobility patterns over long periods of time with a sufficient accuracy for statistical analysis. In this paper, we focus on such system, which is composed of many low-cost visual sensors (shown in Figure 1). Each sensor is equipped with two mouse sensors (30 × 30 pixels resolution sensor used in computer mice) and a digital signal controller. The mouse sensors have actually not been designed to function as camera, but can produce pictures in a debugging mode. The picture quality is far from ideal and the pictures often contain artifacts due to read out problems such as electrical interference. Moreover the mouse sensors do not have embedded common processing steps such as lens shading correction, resulting in significant vignetting. Thus, the image devignetting is performed on digital signal controller as preprocessing steps.

The digital signal controller on each visual sensor is powerful enough to perform all required image processing, even at high frame rates, because of the very low image resolution. They can also communicate with each other over wireless links or over serial cables. When produced in sufficient volumes, the proposed camera network would offer a much cheaper solution than an equivalent network of off-the-shelf cameras, even if the latter were composed of fewer cameras. The proposed system would only be slightly more expensive than sensor networks composed of Passive Infrared Sensors or similar devices, but offers richer data. For instance, it allows rudimentary pose analysis. It can detect not only motion but also presence and it can distinguish people from other moving objects and animals.

A first contribution of this paper is that we show for the first time that reliable people tracking is possible despite the very low resolution of the cameras. A second contribution is that we propose a tracking algorithm that allows all image processing tasks to take place on the digital signal controller in the individual visual sensors. As a result of this distributed processing, image transmission is not needed, making it possible to construct more privacy-sensitive systems. Moreover, the base station that integrates the measurements from the sensors to compute the final tracking results can be quite simple even for networks with many cameras: the base station operates on numbers rather than video streams. It is desirable to keep the low communication load within the camera network to gain benefits such as high scalability, lower power consumption, *etc*. The communication efficiency comparison of our proposed tracker and a state-of-the-art tracker is discussed in Section 5. This distributed Recursive Maximum Likelihood (RML) tracking algorithm proposed herein builds on our earlier work on tracking with high resolution cameras [3] and various adjustments are made to handle the low sensor resolution and the relatively poor image quality (the sensors were not designed for the purpose of video capture).

Many algorithms that perform well under laboratory conditions perform badly in real life conditions or can work only after extensive tuning. In our system these problems are exacerbated by the noisiness of the low resolution images. Moreover, the algorithms need to deal with poor and quickly changing lighting conditions. For all these reasons, it is not trivial to come up with a solution that works in real life circumstances over long periods of time. As a result, classical single image computer vision analysis steps such as feature detection and segmentation perform quite poorly on the video provided by the kilo-pixel sensors. Our solution performs better in this respect, not only because of the details of the image processing (including careful acquisition parameter settings and noise suppression in the sensor), but also because of the use of feedback from the central tracking system. This feedback allows us to use information from other sensors in the image processing on a specific sensor, without actually having to transmit images from the other sensors.

A final contribution of this paper is that we compare our algorithm quantitatively with a reference visual tracking method from literature [4] using ground truth obtained with highly accurate Ultra-Wide Band positioning sensors (note that these UWB sensors are laboratory tools and are too expensive to be suitable for practical deployment). The results show that tracking is feasible with low resolution devices and that our method is more reliable and more accurate than the reference method. Specifically, we provide an extensive report of the performance of our system (calibration, foreground detection, tracking). The results are not perfect, but we show that they are accurate enough to gather useful statistics over longer periods of time. To judge the influence of video processing errors on the overall statistics, we compare our results with the UWB tracking results. This comparison allows us to infer the additional quality gain that could result from further improving video processing up to the point of “perfect” video processing.

This paper is organized as follows. Section 2 describes the related works on different types of sensor networks for Ambient Assisted Living applications and various state-of-the-art visual people trackers. Section 3 gives detailed description of our proposed mobility monitoring system. The evaluation of our proposed system and results are presented in Sections 4 and 5, respectively. Finally, the paper is concluded in Section 6.

## 2. Related Work

People tracking using body-worn devices has many problems, the most prominent one being that people often forget to wear the devices and/or forget to replace dead batteries. For this reason non-body sensors embedded in the environment are often preferred. Passive Infrared (PIR) Motion Sensors offer a cheap and popular solution, but they have some disadvantages: they can only detect objects if they are moving sufficiently quickly and are located within the frustum of the PIR system [5]. Besides, Cardinaux *et al.* [6] state that many PIR sensors are required to track people in a complete room.

The main disadvantage of PIR sensors is that they can only determine the presence moving objects within their frustum. They cannot distinguish between individuals or even between people and animals, and they cannot distinguish even simple actions such as falling *versus* sitting. Vision-based networks use cameras to track people and to analyze their activity and could potentially provide a much richer analysis of their behavior, in addition to tracking their movement.

Wang *et al.* [7] construct a network of six cameras (resolution of 320 × 240 pixels and frame rate of 25 fps) for abnormal behavior detection in outdoor environment. Their work is only evaluated on two short sequences with the duration of 20 and 10 s to detect two abnormal behaviors; running and overtaking. Rowe *et al.* [8] present FireFly Mosaic, a wireless sensor network that has been deployed in an apartment for activity analysis with eight cameras with 352 × 288 pixels resolution. They demonstrate that their system is able to recognize various regions of the house where particular activities frequently occur. However, their approach directly clusters the regions with activities, *i.e.*, average foreground detection by Gaussian Mixture Model (GMM), in image domain instead of tracking a person first.

While cameras have definite advantages over PIR-sensors and related devices, they also have many drawbacks. For instance they are quite privacy-intrusive and they are costly, not so much because of the camera cost, but rather because of the cost of the associated video transmission and processing. In this paper, we study low resolution cameras with embedded video processing as a compromise between PIR-like sensors (very cheap but no detailed analysis possible) and regular cameras (expensive with highly detailed analysis). While these cameras cannot offer the same level of detail in video analysis as high resolution cameras, they still are capable of rudimentary pose analysis.

Low resolution cameras have been considered before in literature. Downes *et al.* [9] design an integrated sensor node for wireless sensor networks and they demonstrate the use of their sensor with a single sensor node with 30 × 30 pixels resolution camera to estimate the direction and speed of persons passing a walkway at the frame rate of 5 fps. However, the goal of low resolution sensor network here in our proposal is to produce trajectories of persons, from which more mobility statistics can be derived.

An important aspect of behavior monitoring is tracking people inside the house, e.g., to determine how often and how easily they move around. There are two major approaches in visual people tracking. The first approach detects people in multiple video frames and then links the individual detections over time [4,10–12]. Jiang *et al.* [10] propose a linear programming relaxation scheme to track multiple persons simultaneously. Zhang *et al.* [11] find the globally optimal trajectories of persons found by the human detector of Wu *et al.* [13]. The multi-camera tracking system of Berclaz *et al.* [4] first utilizes the concept of probabilistic occupancy mapping to find the persons' positions. Then the known positions of each person are linked using the k-shortest path algorithm. The aforementioned systems need an input of the whole video sequence or a batch of frames. This limits their feasibility for online tracking, which requires no significant delay. On the other hand, as stated in [14], these methods can potentially perform better because they can exploit information from future videos frames while determining a person's position.

Other trackers recursively update the tracks from preceding frames with the detections of either people or foreground blobs in the current frame and as such have “video processing in the loop”. In [15] the authors first track people in each camera separately and then integrate these results using a Bayesian approach and relying on the principles of epipolar geometry. Bredereck *et al.* [16] first detect persons using the detector of Dalal *et al.* [17] and Felzenszwalb *et al.* [18] in each camera view and then track these detections with a particle filter within each view. The same people in different views are associated by triangulation, using a greedy matching approach. Their technique relies on color features, which we cannot use since our cameras produce grayscale images. The technique in [19] can work in grayscale sequences as it is based on optimizing the likelihood of foreground/background segmentation images given a hypothesized position in 3D space. The actual data fusion involves a Kalman filter. The method is able to track multiple persons in real-time, but because of the Kalman filter it sometimes loses people when they suddenly change direction.

Our tracker fits in the category of recursive techniques with video processing in the loop. We employ recursive estimation of person's positions based on positions estimated in a previous frame and a uniform motion model rather than dynamic programming over a very large number of past and future observations. Despite the simplicity of our approach, we will show that it outperforms a more complex state-of-the-art approach that also uses information from future video frames and thus introduces a tracking delay. Moreover, systems with “video processing in the loop” are capable of tracking in an online manner, *i.e.*, without significant delay. While we do not study this use case in this paper, such trackers offer the possibility of event detection (e.g., intrusion or fall detection) and therefore are to be preferred regarding overall versatility of the system. In this paper we consider the problem of tracking on low resolution cameras. Even with high resolution cameras, visual tracking of people in an uncontrolled environment is still very challenging as the appearance of a person changes with body movements, changes in pose and orientation, and lighting changes. When the resolution of input video is as small as 30 × 30 pixels, the task of people tracking becomes very difficult.

While many visual tracking algorithms have been proposed for tracking in high resolution cameras, few papers deal with some important aspects relevant for our work: operating well on low resolution images, operating in real time and working well over long periods of time in diverse environmental circumstances. Most of the aforementioned trackers have been tested only on medium/high resolution videos but can of course be applied to low resolution video. Gruenwedel *et al.* [20] demonstrate that an occupancy mapping based tracker still works reasonably well on video with a resolution as low as 64 × 48 pixels. However, this low resolution video is *simulated* by low pass filtering and downsampling high resolution video and is quite different in quality than the video we process in this paper.

In Section 5 we will compare our own tracker to the occupancy mapping based tracker by Berclaz *et al.* [4], which we tuned for low resolution videos and which seemed to perform the best among other available high resolution methods we tested in a preliminary experiment.

## 3. The Proposed Mobility Monitoring System

**Figure 2.**
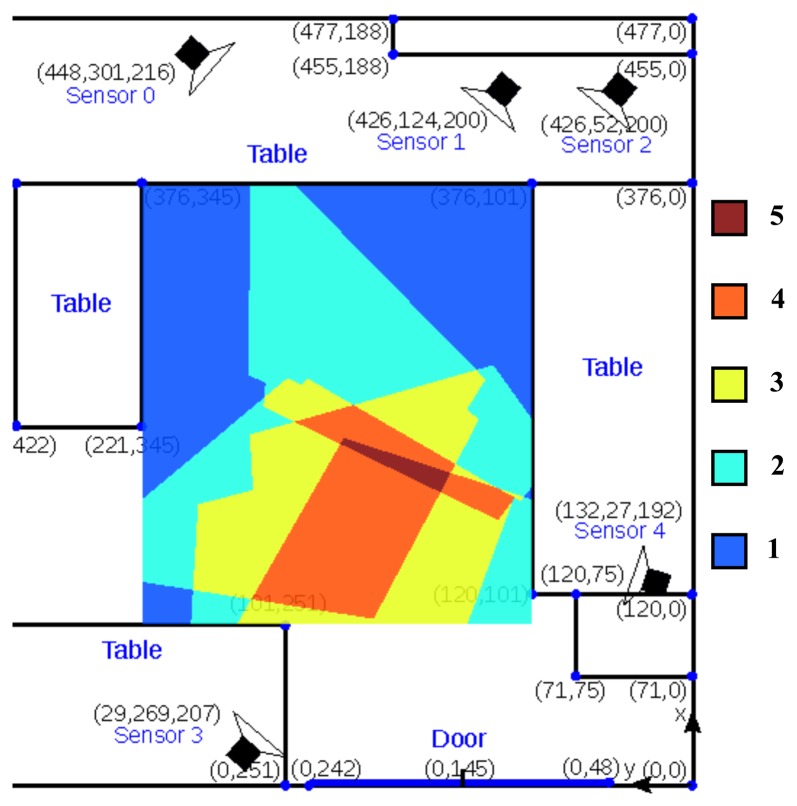
Room layout showing the configuration of five visual sensors (each containing stereo mouse sensor) covering an area of 5 × 5 m^2^. The colors indicate the number of cameras observing a specific point on the ground plane.

The results in this paper were obtained on an initial system setup in a relatively small room (5 × 5 m^2^) in our lab. Figure 2 shows a floor plan of the setup. This system consists of five stereo visual sensors. The placement of these sensors is non-ideal because of practical constraints. Figure 2 displays the visual sensor coverage of the room. Most of the possible walking area is covered by at least two sensors, except the upper left and upper right regions near the table, which are covered by only one sensor. The effect of camera coverage on the performance our tracker is discussed in Section 5. The main goal of this system was to test the hardware and tracking algorithms before installing a larger test system with 10 sensors in larger service flats, where it will be used to monitor the behavior of elderly people over many weeks. In fact the system has very recently been installed and will be operational soon.

Currently the sensors are connected to two nearby PCs using serial cables. All data analysis proceeds on these PCs. However, in the envisaged final system, all video processing will be run on the digital signal controller embedded in the visual sensors; the visual sensors will be connected to a micro PC that will act as a fusion center. The fusion center then only performs data fusion and broadcasts the fused result (the coordinates of the bounding boxes of the tracked persons) back to all sensors. This feedback, although simple in principle, is a crucial component of the tracking system.

With some changes to the tracking algorithm (future work), we expect it will be possible to construct a system requiring only low bandwidth communication between the sensors and the fusion center. Therefore, the final system will employ wireless communication. Given the low power consumption of the sensors, we will also be able to operate them on battery over prolonged periods of time. Hence the final system will not require any rewiring in the service flats.

On the software side, the monitoring system consists of two layers: the bottom “tracking” layer that tracks people, and the top “analysis” layer that analyzes the tracks and converts them into useful statistics. In this paper we focus on the bottom layer, but we evaluate its performance taking into account the requirements of the top layer. For instance, we can accept a certain level of tracking errors as long as the computed statistics remain sufficiently accurate.

**Figure 3.**
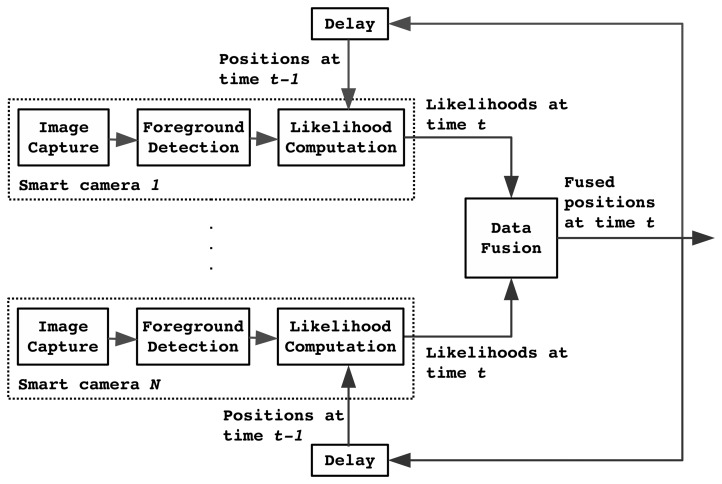
System architecture of our proposed tracker.

Figure 3 shows a block diagram of the recursive tracker, which operates at 33 fps. One analysis cycle for processing a single video frame roughly proceeds as follows. First, each sensor captures a new frame and performs preprocessing on this frame (denoising, devignetting, automatic gain control). Then, the frame is analyzed to separate moving objects from the static background. This results in a number of blobs, not all of which correspond to actual persons due to imperfections in the video processing and because of moving objects that are not of interest, such as chairs.

Each blob is checked if it is overlapping with the bounding boxes of the tracked persons in the preceding frame. Only non-overlapping blobs are considered as candidate blobs for detecting a new person to be tracked. Firstly, the smallest possible bounding box for each candidate blob is determined. If there is no occlusion, the position of the person's feet in image coordinates can be approximated as the center of the bottom edge of the bounding box. The approximated feet position in image coordinates can be converted into position on ground plane in world coordinates using homography.

Only approximated feet positions in world coordinates of the candidate blobs are sent to the fusion center. The candidate positions of one sensor are matched with the candidate positions in other sensors. Due to the presence of noise in foreground blobs and differences in calibration accuracy for each sensor, the candidate feet positions estimated by different sensor may not project to the same point in world coordinates. However, they will be close to each other on the ground plane. Thus the matching criterion is defined as thresholded Euclidean distance rather than an exact position match. If a candidate position of one sensor is matched with the candidate positions of other *N* or more sensors, a new person to be tracked is initialized at the centroid of all matched candidate positions.

Next, each sensor analyzes the likelihood that a person is in specific position in the room, given the current foreground image and knowledge of the location of persons in the preceding frame. The fusion center then fuses these likelihoods and estimates the most likely new position of the person. Finally, the jointly estimated position is fed back to all sensors, which will use this information when processing the next frame.

In the following *t* will be the frame number. In the experiments we will consider only a single person. This is sufficient for our initial use case (a person living alone; the analysis can be suspended when a visitor is present). Our tracker can handle multiple persons in principle, but the results below are restricted to a single person. The position in image coordinates of a reference point on the tracked person *m* will be denoted by **r** = (*i*, *j*)*^t^*; the person's position on ground plane in world coordinates will be denoted by **s** = (*x*, *y*)*^t^*. Since visual sensors are calibrated, **s** can be projected onto image plane **r** = *P_c_***s**, where *P_c_* is a projection matrix of a visual sensors *c*. Below, we discuss some aspects of the system in greater detail.

### 3.1. Video Capture and Pre-Processing

The low resolution visual sensor contains two Agilent ADNS-3060 high-performance optical mouse sensors, popular for gaming applications. These sensors are used as motion detectors in normal mouse operation: firmware in the sensors that detects feature points in the images and tracks them over time. The sensors typically operate at a programmable frame rate of about 2000 to 6000 frames per second. We operate the sensors in debugging mode, which allows capturing images of a resolution of 30 × 30 pixels and an image depth of 6 bit per pixel. This way, we can operate the sensors as low resolution cameras. This approach has quite a few restrictions.

Firstly, the debugging mode only allows capturing a single image, after which a delay of over 3 frames must be respected before capturing the next image. As such, at most one quarter of the light reaching the sensor can actually be captured as images. Moreover, the sensor is designed for very high frame rates (2000 to 6000 fps), whereas the debugging mode only allows reading the image data at a maximum frame rate of about 100 fps due internal bandwidth limitations. At an internal frame rate of 2000 fps—the lowest possible according to the datasheet—the sensor would only capture about 5% of the incoming light.

Fortunately, it is possible to operate the sensor far beyond the manufacturer's specifications, which allows internal frame rates as low as 400 fps. In addition, the sensor's pixels have a large surface area and therefore capture more light than those of high resolution cameras. Still, the images are quite noisy and because of the limited grayscale resolution, they have a low dynamic range and tend to display strong contouring in flat areas.

Our visual sensor is also equipped with an embedded digital signal processor featuring a 16-bit wide data path and 64 kB RAM: a Microchip dsPIC33FJ128GP802 digital signal controller. Currently this digital signal controller is used to handle image preprocessing and image transmission. Although the results in this paper are built on PC-based video analysis, the digital signal controller is actually powerful enough to perform the video analysis (foreground/background segmentation, blob extraction, *etc.*) required by the tracking algorithm. This is important, because it means that our tracker will eventually be able to operate without the cameras having to transmit video, which will result in a privacy-friendly solution.

The first step of image preprocessing is denoising: a simple time recursive filter averages the gray values of each pixel over time, thus reducing noise. In order to produce a sharp image of the outside world, a lens needs to focus light properly on the imaging sensor. Lenses typically cause an effect called “vignetting”: the strength of the light projected by the lens onto the sensor is amplitude modulated according to a pattern of concentric circles. The second step of preprocessing, devignetting, compensates for this problem and also corrects any pixel-dependent dark stream current in the mouse sensors.

Because of the low sensor integration time (see above: at 400 fps, the pixels integrate light over only about 2.5 ms) the captured video also suffers from another problem, which is quite prominent indoors: intensity fluctuations due to fluorescent lights. These lights flicker (in Europe) at a frequency of about 100 Hz. If the camera is operated at a frame rate not harmonically related to this flicker frequency, the captured video will display unwanted intensity variations over time. Typically we operate the camera at frame rates of 25 fps, 33.3 fps or 50 fps. However, as the digital signal controller's clock is not synchronized with the mains frequency, these frame rates are *almost but not exactly* harmonically related to the flicker frequency. As a result, and without further measures, this results in a quite noticeable low frequency variation in light intensity with a period of typically a few seconds.

Most of the results in this paper have been obtained on such “light modulated sequences” and apparently the foreground/background segmentation algorithm of the methods in our results section are robust enough to handle this effect.

However, we have been able to solve the light modulation problem without resorting to complex control loops: by carefully timing image acquisition, it is possible to increase the period of the light modulation to such a degree that the sensor's automatic gain control can compensate for it. This requires careful tuning of the digital signal controllers' main clock frequency. As the main frequency is quite stable in Europe, this simple approach allows a stable solution over long periods of time.

### 3.2. Correlation-Based Foreground/Background Segmentation

Foreground/background segmentation locates moving objects in the scene by comparing the current input frame *I*(**r**; *t*) with a reference frame *I*_ref_ (**r**; *t*). For this purpose we have adapted the algorithm we developed earlier for high resolution cameras, which was shown to be quite robust to illumination changes [21]. This means it is almost not affected by the light flicker issue. More importantly, robustness to quick and slow illumination changes is essential in a home environment, where lights may be switched on or off at any time. While the automatic gain control in the sensors can to some degree compensate for *overall* light changes, it cannot compensate for local light changes, e.g., when half of the room becomes more strongly illuminated.

The employed foreground/background segmentation method analyzes changes in image structure (e.g., edges in the scene) between two images, rather than changes in gray value as most existing methods [22,23] do. The reference background image *I*_ref_(**r**; *t*) is initialized as the time average of a short video sequence captured while no persons are in. It is then slowly adapted over time (see below).

Changes in *I*(**r**; *t*) with regard to *I*_ref_(**r**; *t*) are detected by computing the following correlation coefficient per pixel using a sliding window approach:

(1) $$\rho (r;t)=\frac{{\text{∑}}_{r^{\prime }\in w(r)}I(r^{\prime };t)I_{\text{ref}}(r^{\prime };t)}{\sqrt{{\text{∑}}_{r^{\prime }\in w(r)}I{\left(r^{\prime };t\right)}^{2}{\text{∑}}_{r^{\prime }\in w(r)}I_{\text{ref}}{\left(r^{\prime };t\right)}^{2}}}$$

where *w*(**r**) is a square window with size *k*. Denote *i_w_* and *j_w_* as zero-based row and column indexes of *w*. For all odd size windows, the anchor point of *w*, *i.e.*, a point on *w* that corresponds to pixel position *r*, is at $i_{w}=j_{w}=\frac{k+1}{2}$. If the window size is even, the anchor point position is at $i_{w}=j_{w}=\frac{k}{2}$. A pixel at position r is considered foreground if *ρ*(**r**; *t*) < *ρ_min_* and background otherwise. In the following, *F*(**r**; *t*) will be the resulting binary image in which the foreground pixels are those with value *F*(**r**; *t*) = 1.

**Figure 4.**
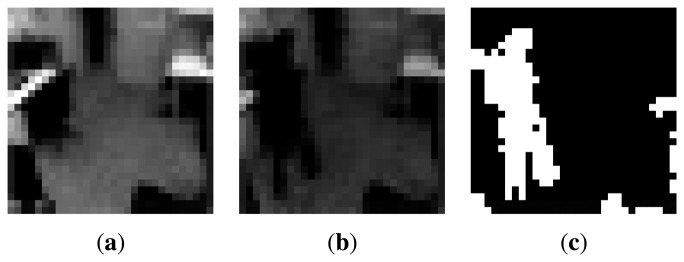
An example (**a**) background image *I*_ref_(**r**; t); (**b**) input image *I*; (**c**) detected foreground image *F*.

Figure 4 shows an example of a reference image *I*_ref_(**r**; *t*), an input frame *I*(**r**; *t*) and the corresponding foreground image *F*(**r**; *t*). In order to reduce the number of false foreground pixels, the background image I_ref_(**r**; *t*) is updated so that displaced non-human objects incorporate slowly into *I*_ref_(**r**; *t*). The background image *I*_ref_ (**r**; *t*) is slowly updated recursively as follows:

(2) $$I_{\text{ref}}(\text{r};t+1)=(1-\alpha )I_{\text{ref}}(\text{r};t)+\alpha I(\text{r};t)$$

where *α* is the learning factor. We experimentally find that a sliding window size of 2 × 2 pixels with *ρ_min_* = 0.98 and *α* = 0.005 gives the best system performance.

### 3.3. Likelihood Model

As before, let s be a possible person location on the ground plane in world coordinates. Each visual sensor estimates the likelihood *l*(**s**; *t*) that position s is occupied by person *m* at time *t*, based on its foreground image *F_c_*(**r**; *t*) and the last known position **ŝ***^m^*((*t* − 1)) of that person; the latter is in fact the fused position fed back from the fusion center. The last known position is used only to restrict the range *R*(**s**) in which to find the new position **ŝ***^m^*(*t*) of the person, *i.e.*, it restricts the values of s for which we compute likelihoods below (see Section 3.4). In Bayesian terms, this means that we assume a uniform prior for s in a square region *R*(**s**; (*t* − 1)) centered on **ŝ***^m^*((*t* − 1)).

In our approach, we assume that the tracked person will always fit within a fixed-size cuboid. For each possible location s this cuboid is projected into the image coordinates using projective geometry and the known camera calibration matrices. In the following we denote by Ω*_c_*(**s**) the visible part of the projection of the cuboid in the sensor's image. Any part of the cuboid outside the sensor's frustum does not contribute to Ω_c_(**s**); specifically, if the cuboid is fully outside the sensor's frustum, Ω_c_(**s**) will be the empty set.

As in [4,14], we propose a model for the likelihood of a camera observing a given foreground image *F_c_*(**r**; *t*) when the true position of a person is s: Ideally, if a person is at location *s* and no other persons are in the picture, then Ω*_c_*(**s**) must contain all foreground in the image. If multiple persons are presented, the union of their projected boxes must contain all foreground.

In practice the ideal situation is never reached and we need to adopt a statistical model. In this paper, we rather propose a likelihood based on the “noisy binary channel model”, which assumes that any foreground pixel can be turned into a background pixels and vice versa due to noise and other problems with a small pixel-independent probability.

Furthermore, we assume that conditioned on s all pixels in *F_c_*(**r**; *t*) in the foreground image are statistically independent. As a result, the overall likelihood of *F_c_*(**r**; *t*), *i.e.*, the conditional probability *P*(*F_c_*|**s**) of the foreground image *F_c_*, has a binomial distribution. Let *ϵ_f_* be the probability that a true foreground pixel is accidentally detected as background and *ϵ_b_* be the probability that a true background pixel is accidentally detected as foreground. Then, given the aforementioned assumptions, the likelihood *l_c_* of a person's position at (*s*) becomes:

(3) $$l_{c}=\prod_{\text{r}\in \Omega_{c}(\text{s})}{\left(1-\epsilon_{f}\right)}^{F_{c}(\text{r};t)}\epsilon_{f}^{1-F_{c}(\text{r};t)}\prod_{\text{r}\notin \Omega_{c}(\text{s})}{\left(1-\epsilon_{b}\right)}^{1-F_{c}(\text{r};t)}\epsilon_{b}^{F_{c}(\text{r};t)}$$

The first product computes how well the pixels in Ω*_c_*(**s**) agree with an hypothesis that a person is at **s**. Similarly, the second term computes how well the pixels outside of Ω*_c_*(**s**) agree with the hypothesized person's location **s**. With log likelihood, Equation (3) simplifies to

(4) $${\text{ll}}_{c}(\text{s})=\sum_{\text{r}\in \Omega_{c}(\text{s})}\left(F_{c}(\text{r};t)\ln\left(\frac{1-\epsilon_{f}}{\epsilon_{f}}\right)+\ln(\epsilon_{f})\right)+\sum_{\text{r}\notin \Omega_{c}(\text{s})}\left(\left(1-F_{c}(\text{r};t))\ln\left(\frac{1-\epsilon_{b}}{\epsilon_{b}}\right)+\ln(\epsilon_{b}\right)\right)$$

If we denote the area of a set *S* as |*S*|, the area of the complete image as |*A*| and we introduce the notations $\lambda_{f}≜\ln\epsilon \frac{1-\epsilon_{f}}{\epsilon_{f}})$, $\lambda_{b}≜\ln\left(\frac{1-\epsilon_{b}}{\epsilon_{b}}\right)$ and $\lambda ≜\ln\left(\frac{\epsilon_{f}}{\epsilon_{b}}\right)$, then Equation (4) simplifies to

(5) $${\text{ll}}_{c}(\text{s})=k+\lambda \left|\Omega_{c}(\text{s})\right|+\lambda_{f}\sum_{\text{r}\in \Omega_{c}(\text{s})}F_{c}(\text{r};t)+\lambda_{b}\sum_{\text{r}\notin \Omega_{c}(\text{s})}\left(1-F_{c}(\text{r};t)\right)$$

where *k* ≜ |*A*| ln(*ϵ_b_*) is a constant independent of **s**. The interpretation of this equation is simplest when *ϵ_f_* = *ϵ_b_*, *i.e.*, when the channel model is symmetric. In this case, the equation shows that the log-likelihood increases when more foreground pixels occur in the projected cuboid and when fewer foreground pixels occur outside the projected cuboid. As previously mentioned, because of the non-ideal placement of sensors due to practical constraints, only a small region in the middle of the room can be seen by all five sensors. Thus, for a given position in world coordinates, s may be observed only by a subset of sensors. When s cannot be observed by a specific camera c, the projected cuboid is completely outside of the image, *i.e.*, Ω(s) is empty. Thus the first sum in Equation (5) becomes zero and ll*_c_*(s) is reduced up to a constant, *i.e.*, ll*_c_* of a particular camera *c* is constant for all s outside of its field of view (FOV).

### 3.4. Data Fusion

In our earlier work [19], our tracker relied on Kalman models to provide a prior probability on the new (unknown) position **ŝ***^m^*(*t*). Basically, these models predict the new position based on earlier estimates: positions close to this predicted position are assigned a high prior probability and positions far away from it a lower probability. The underlying models are Gaussian. We noticed that this solution performs poorly in the case of abrupt changes in speed. Moreover, the optimal Kalman gain depends on the actual speed of the person.

In this paper we adopt a *random walk* motion model. We assume that in the time between frames (*t* − 1) and *t* the person can move anywhere within a fixed size region *R*(**ŝ***^m^*((*t* − 1))) centered on the old known position **ŝ***^m^*((*t* − 1)) and that all positions are equally likely. This corresponds to a uniform prior within *R*(**ŝ***^m^*((*t* − 1))). The maximum a posteriori estimate of **ŝ***^m^*(*t*) is then found simply by maximizing the sum of the likelihoods computed by all cameras, in which *k′* is an unimportant constant:

(6) $${\hat{\text{s}}}^{m}(\text{t})≜\underset{\text{s}\in \text{R}(\text{s};t-1)}{\max}\text{ll}(\text{s})$$

where

(7) $$\text{ll}(s)≜\sum_{c\in C}{\text{ll}}_{c}=k^{\prime }+\lambda \sum_{c\in C}\left|\Omega_{c}(\text{s})\right|+\lambda_{f}\sum_{c\in C}\sum_{\text{r}\in \Omega_{c}(\text{s})}F_{c}(\text{r};t)+\lambda_{b}\sum_{c\in C}\sum_{\text{r}\notin \Omega_{c}(\text{s})}\left(1-F_{c}(\text{r};t)\right)$$

where *C* is the set of cameras used, and the search range is restricted to **s** ∈ *R*(**ŝ***^m^*((*t* − 1))).

The interpretation of this equation is the following: the fusion center chooses s such that the total number of foreground pixels in the projected cuboids is as high as possible and the total number of background pixels outside these projected cuboids is also as high as possible. The constants λ*_f_* and λ*_b_* control the relative importance of both criteria. The term with the factor λ modulates the log-likelihood by attaching more or less importance to cameras closer to the person. This term is only relevant if *ϵ_f_* ≠ *ϵ_b_*.

Due to the uniform prior for the motion model, the computations are simpler and more robust in our tracker than in methods relying on more complex priors. The motion model is also incorporated into the recursive likelihood computation, whereas in some of the state-of-the-art trackers, it is used only in subsequent post-processing. An important practical benefit of our approach is that it is much more suitable for distributed processing. In fact, each ll*_c_* in Equation (7) can be computed in camera *c*. Afterwards, only the computed values ll*_c_*(**s**), for **s** ∈ *R*(**s**; (*t* − 1)), need to be sent to the central fusion center, which can be done quite efficiently.

**Figure 5.**
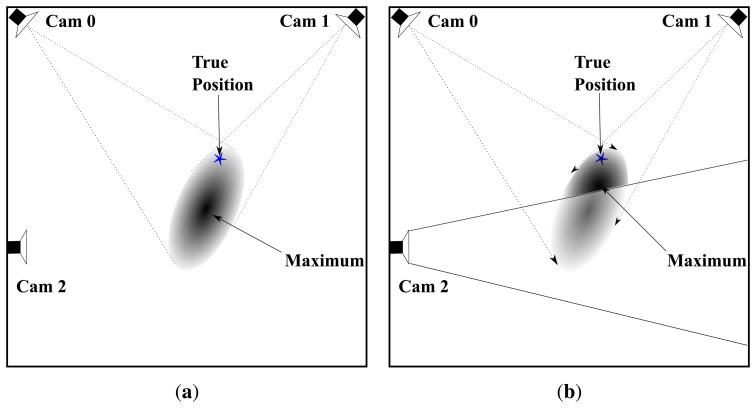
Joint likelihood computed using (**a**) camera 0 and 1; (**b**) camera 0, 1 and 2. Darker color indicates higher likelihood.

All sensors (5 stereo visual sensors for our setup) are included in the calculation of ll(**s**) even when s is not in their FOV. Despite the non-visibility of a person in the FOV of a particular camera *ĉ*, including likelihood ll*_ĉ_* in the calculation of ll results in a more accurate joint estimate. Figure 5 demonstrates this with a toy example with three cameras. Suppose a person is at the location marked by a blue star, which is in the FOV of camera 0 and 1 but not in the FOV of camera 2. Figure 5a shows the joint likelihood computed from the observed foreground image of camera 0 and 1. However, the position of the likelihood peak is a bit far from the true position (probably due to noise/error in foreground detection). When camera 2 is included in the joint likelihood computation, it contributes a term to the likelihood, which reduces the overall likelihood within its FOV since it does not observe any foreground pixels. This shifts the likelihood peak position closer to the true position of a person.

Our tracker handles occlusion based on the assumption that the tracked person is not occluded in at least one sensor view within the camera network. This assumption is usually valid in practice for visual sensor network with overlapping views, which sees the target from different view angles. Given this worst case that a person is not occluded in only one sensor view, ll(s) given by Equation (7) will be maximum at a position **ŝ***^m^*(*t*) where the log-likelihood *ll_c_*(*s*) computed from non-occluding sensor view using Equation (5) is maximum.

## 4. Performance Evaluation

### 4.1. Datasets

For validating the performance of our proposed tracker, we captured two multi-camera sequences of 30 min duration each. In this experiment, a single person was asked to walk around in the room and sit at different seats from time to time, while carrying a UWB receiver. In both sequences, a person walks in the room several times for approximately 30% of total during of the video. The video and the UWB data were time-synchronized. The video frame rate was 33 fps. The UWB produced data at a rate of 2.5 Hz.

The UWB testbed comprises six PulseOn P410 UWB ranging devices [24], of which five were used as fixed anchors and one as the mobile terminal, which was connected to a digital signal controller. These devices can achieve a spatial resolution up of about 3 cm (RMSE) by transmitting and analyzing radio waves in the spectral range from 3.1 GHz to 5.3 GHz [24]. To enable real-time positioning, the low-complexity linear least squares algorithm [25] was used for position estimation. In the post-processing step, we eliminated outliers by discarding all inconsistent location estimates (*i.e.*, a residue larger than 5 m^2^). In this paper, the UWB is used as ground truth data to estimate the accuracy of the visual tracking systems only. The UWB devices are not practical enough for actual applications.

**Figure 6.**
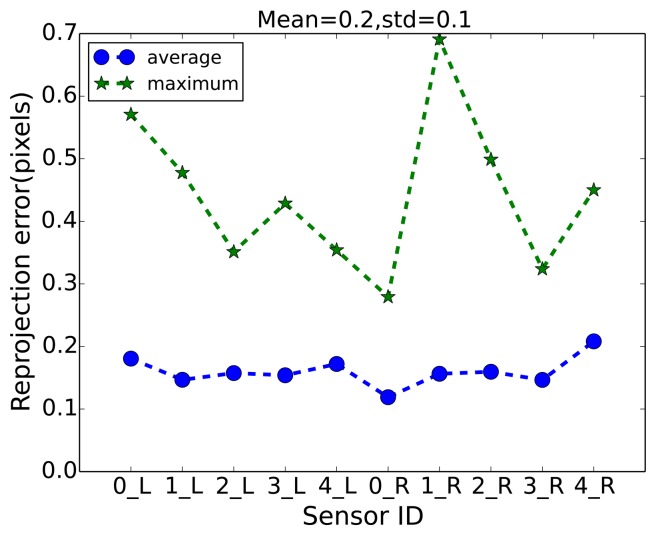
Reprojection error for each visual sensor.

The intrinsic parameters for all visual sensors used for capturing the aforementioned multi-camera sequences are obtained using the calibration method proposed by Zhang [26]. The extrinsic parameters were estimated by the method proposed by Guan *et al.* [27], which uses a sphere as a calibration object. The calibration accuracy, *i.e.*, reprojection error, of all visual sensors are shown in Figure 6. The reprojection errors were computed on a number of spheres positioned in the room. They are defined as the mean square-root distance between the observed pixel coordinates of the spheres and the pixel coordinates computed by projecting the known world coordinates of the sphere centers into the images.

### 4.2. Evaluation Criteria

The overall mobility of a person can be assessed using simple statistics, such as average and maximal walking speed, total walking distance (per day) and total walking time. However, in our future work we would like to focus on finer and higher level analysis, e.g., by computing mobility parameters for specific types of trajectories. For instance, trajectories from the TV area to the kitchen may take longer because of problems after sitting still for prolonged periods.

In this paper we assess if the tracking system is sufficiently accurate and reliable to support this type of analysis. In general and qualitatively we consider two criteria: (1) tracks are considered invalid if they do not correspond to a real person or if they deviate significantly from the true persons position over prolonged periods of time; (2) for valid tracks only, the accuracy of the track is of importance, *i.e.*, on average how close is the tracked position to the true position. A track is considered valid if its Total Average Tracking Error (TATE) is below threshold *T*. Otherwise, it is regarded as a bad track. TATE is simply the average of the Euclidean distances between positions estimated by the tracker and the corresponding positions given by the UWB testbed.

While TATE shows the average accuracy of the tracker over the whole video sequence, the distribution of the errors is also important. For instance in some applications, large errors may be intolerable whereas smaller errors are irrelevant. For this reason, we also analyze the number of *tracking losses*; we consider tracking is lost when the Euclidean distance between the estimated position and the ground truth point exceeds a specific threshold *T_loss_*. The Percentage Tracking Loss (PTL) of a sequence is computed as the percentage of the total number of frames with tracking loss in the total number of frames compared with the ground truth.

## 5. Results and Discussion

The performance our proposed Recursive Maximum Likelihood (RML) tracker is evaluated by comparing it with the UWB ground truth. Note that the UWB ranging device needed to be carried by the test person. For this reason the UWB position is always close to but never “on” the person. This leads to a systematic offset of around 10 cm with regard to the visual tracking result. Moreover, when the person was sitting, the UWB device was placed on the table. These effects need to be taken into consideration when judging the results. Not only the UWB estimates but also the estimates of both trackers in comparison sometimes fall in the area where a person cannot walk on (for example, on the table). Since the regions in the room in which a person cannot walk on is known, the estimates (of whether UWB or trackers) found in those non-walkable regions are shifted to nearest walkable area.

We also compare the proposed RML tracker to the tracker of Berclaz *et al.* [4]. Their approach approximates a posterior conditional probability of occupancy, the so-called Probabilistic Occupancy Map (POM), on the ground plane using conditional likelihood model built from the result of foreground/background segmentation in all camera views. Since their POM computation only considers information from the current frames of all cameras but not the positions estimated in the previous frame, the quality of the initial POM is insufficient. This is solved by using iterative procedure, which significantly increases computation time. However, in the data association step, the *K*-Shortest Path (KSP) optimization is used to find the optimal trajectory of a person by enforcing temporal continuity constraints over POMs computed from both past and future frames. We will refer to their tracker as POM-KSP. We tune the parameters for POM-KSP tracker by experimentlly selecting the paramter values which give the lowest TATE from different combinations of vaules around default parameter values reported in [4].

**Figure 7.**
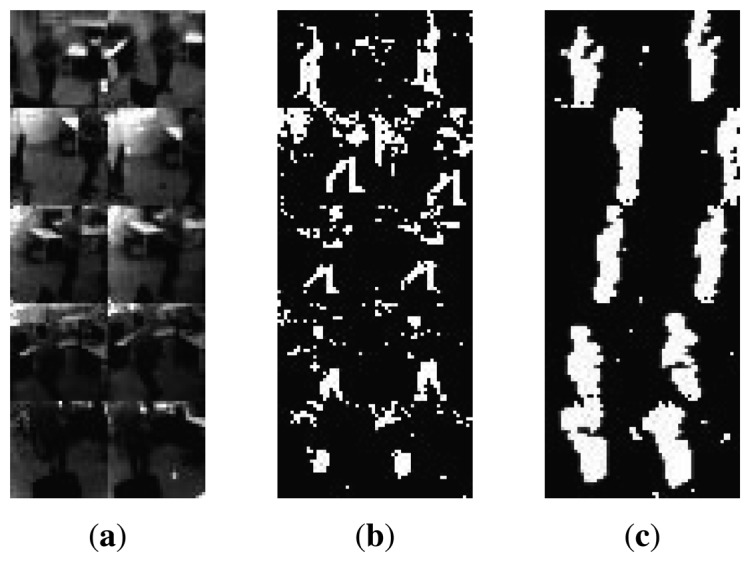
Left to right: (**a**) original image; (**b**) foreground detection by ViBe; (**c**) foreground detection by our correlation-based method.

For our dataset, most of the existing methods already have problems in the initial stages of video processing, *i.e.*, the robust segmentation of foreground objects (people) from the scene background, due to the aforementioned intensity fluctuation problem. Specifically, methods like [22,23] react poorly or too slowly to lighting changes. Figure 7b,c shows the foreground/background segmentation results of optimally tuned ViBe [23] and our method based on correlation. Visual inspection clearly shows that the ViBe method fails to detect approximately 40% of the person's body with noticeably high number of false detections in the background. However, our correlation-based method is able to detect almost 100% of the person's body with relatively lower number of false detections.

Table 1 shows the result of the experiment. The TATE values of our proposed RML tracker are compared with those of Berclaz *et al.*'s POM-KSP tracker. In our current track, the random walk model only imposes some weak constraints on the temporal continuity of the tracks. While this property is essential to avoid tracking loss, it is not ideal regarding the track accuracy. For this reason, Table 1 includes not only results for the “raw” tracker output, but also results after applying simple forms of sliding window post-processing: temporal median and temporal mean filtering. In both cases the sliding window size was 5.

Clearly our RML tracker outperforms the POM-KSP tracker in accuracy (TATE scores) both with and without post-processing. Post-processing improves the results for both methods, but not spectacularly so. Table 1 also shows the number of good/bad tracks for both methods in both datasets. As the mean filter produces the best result for both trackers, we only consider the tracks post-processed by it in this analysis. Our definition of a good track is that the TATE score should be less than 60 cm. This threshold is chosen because it is about the minimum distance between two people in most situations of interest.

**Figure 8.**
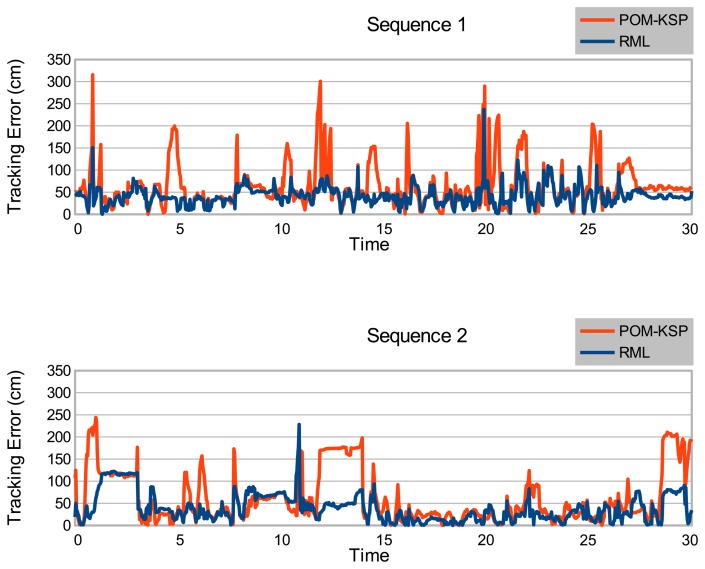
Tracking error (TE) for all compared frames.

Figure 8 shows the tracking error (TE) for all compared frames of both Sequence 1 and 2. In both plots, the tracking error of our proposed tracker remains under 100 cm for 93.5% of the total compared frame. Even when the tracking error exceeds 100 cm, it drops again almost immediately. In contrast, the tracking error of Berclaz *et al.*'s tracker exceeds 100 cm more often and it takes a while to drop back again (takes longer time to recover from tacking loss). To our knowledge, trapping in local minima of k-shortest paths optimization of Berclaz *et al.*'s tracker causes this prolonged tracking loss. Table 2 shows the Percentage Tracking Loss (PTL) of our proposed tracker and the tracker of Berclaz *et al.*, for different *T_loss_* values. In both sequences, our tracker outperforms for all *T_loss_* values. The choice of *T_loss_* depends on the application and evaluation goals.

**Figure 9.**
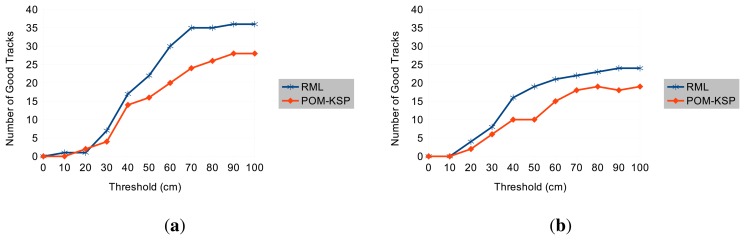
Dependency of the number of good tracks (after mean filtering) on the selected threshold. (**a**) Sequence 1; (**b**) Sequence 2.

**Figure 10.**
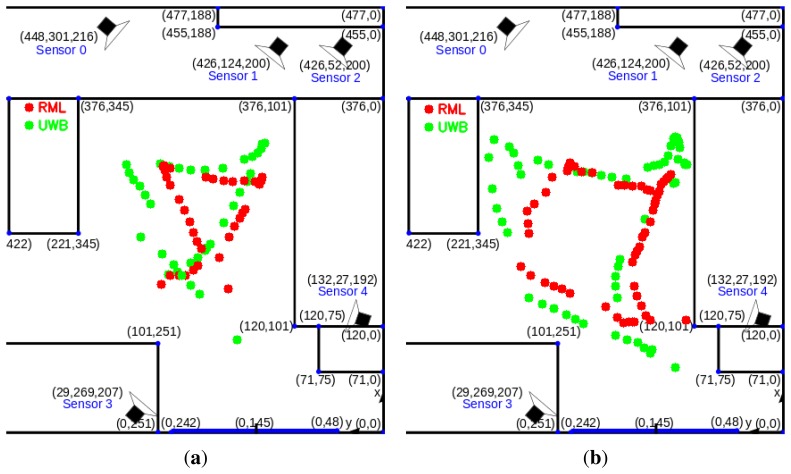
Comparison between RML tracks (blue) and UWB tracks (green). (**a**) Track 1; (**b**) Track 2.

Table 1 shows that the RML tracker produces 2 to 3 times fewer bad tracks depending on the dataset. Figure 9 shows how the number of good tracks varies with the selected threshold. Interestingly at very rigorous thresholds (when most tracks are rejected), POM-KSP performs slightly better than RML. However, at this threshold most tracks are invalid in both methods. This can be attributed to the very low resolution of the cameras and the associated limitations in image processing and calibration. Figure 10 shows some example of good tracks obtained by RML and UWB. The comparison in Figure 10 indicates that the shape of tracks, *i.e.*, walking pattern of a person, produced by RML and UWB are very similar despite small deviation in their location estimates.

Speed and distance measures are calculated per track for the RML tracker and the UWB data points. The distance traveled d in each track is computed as

(8) $$d=\sum_{n=2}^{N}\left\|\text{s}(n-1)-\text{s}(n)\right\|$$

where *N* is the number of person's positions **s** of a track. Moreover, the duration *T* of a track (in seconds) is calculated:

(9) $$T=\frac{N}{\text{framerate}}$$

where *framerate* is the UWB frame rate. Having the time and distance calculated per track, the speed is defined according to the following relationship between time and distance:

(10) $$\upsilon =\frac{d}{T}$$

**Figure 11.**
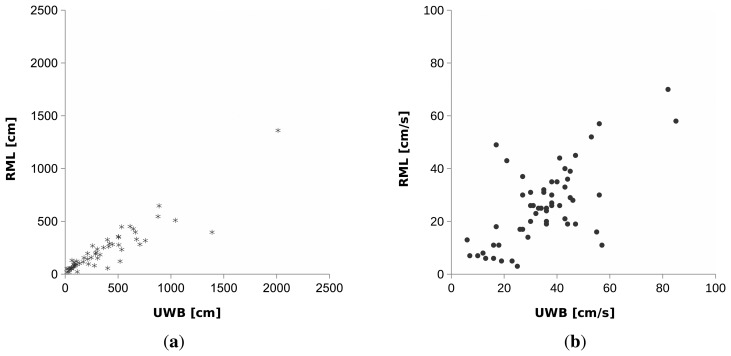
Scatter plot showing correlation between mobility statistics of each track computed from trajectories (both Sequence 1 and 2) of RML and UWB. (**a**) Total distance traveled per track; (**b**) Average speed per track.

It can be clearly seen from the scatter plot in Figure 11a that the distance traveled per track (both Sequence 1 and 2) computed from RML and UWB is highly correlated. The total distance traveled *D* in each sequence is simply the sum of distance traveled of all tracks in the sequence. For Sequence 1, *D* computed from RML and UWB is $D_{1}^{\text{RML}}=72.83\text{m}$ and $D_{1}^{\text{UWB}}=118.93\text{m}$, respectively. The value of D computed from RML and UWB for Sequence 2 is $D_{2}^{\text{RML}}=52.77\text{m}$ and $D_{2}^{\text{UWB}}=52.77\text{m}$. Although there is relatively large difference between *D* computed RML and UWB, the correct statistical inference can still be made from RML. For example, *D* of both RML and UWB suggests that the person in Sequence 1 walks more distance than the person in Sequence 2 ( $D_{1}^{\text{RML}}>D_{1}^{\text{RML}}$ and $D_{1}^{\text{UWB}}>D_{1}^{\text{UWB}}$).

Moreover, the scatter plot in Figure 11b shows that the average speed per track (both Sequence 1 and 2) is well correlated and outliers are few. Most outliers are from short tracks with few position estimates. The average speed *ῡ* of a person in each sequence can be computed by dividing *D* with the sum of the duration *T* of all tracks in the sequence. Tracks of RML and UWB in Sequence 1 give *ῡ* of ${\bar{\upsilon }}_{1}^{\text{RML}}=17.85\text{cm}/\text{s}$ and ${\bar{\upsilon }}_{1}^{\text{UWB}}=29.15\text{cm}/\text{s}$ while Sequence 2 gives ${\bar{\upsilon }}_{1}^{\text{RML}}=12.85\text{cm}/\text{s}$ and ${\bar{\upsilon }}_{1}^{\text{UWB}}=21.15\text{cm}/\text{s}$. From observing *ῡ* given by UWB ( ${\bar{\upsilon }}_{1}^{\text{UWB}}>{\bar{\upsilon }}_{2}^{\text{UWB}}$), it is clear that on average the person in Sequence 1 walks faster than the person in Sequence 2. The same conclusion can be reached from *ῡ* given by RML ( ${\bar{\upsilon }}_{1}^{\text{RML}}>{\bar{\upsilon }}_{2}^{\text{RML}}$).

**Figure 12.**
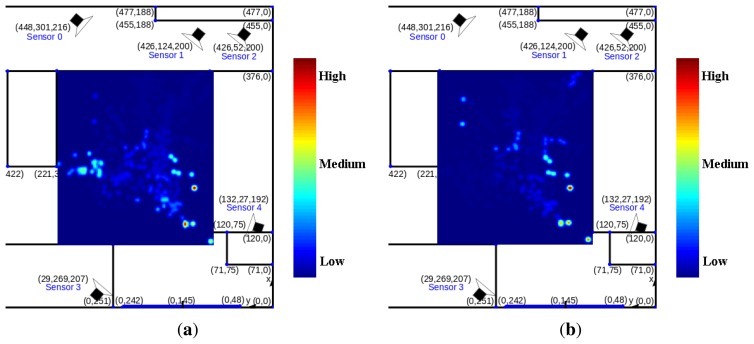
Average speed map computed from trajectories of (**a**) our proposed tracker (RML) and (**b**) UWB.

We also show the average speed calculated for each discretized cell of the room for both RML and UWB in Figure 12a,b. From visual comparison, it can be seen that both maps have similar speed distribution. Moreover, both maps show that people tend to walk faster when they are walking to/from the door. Low speed distributing around the center of the room in both maps suggests that people walk slower when they walk around the center of the room.

**Figure 13.**
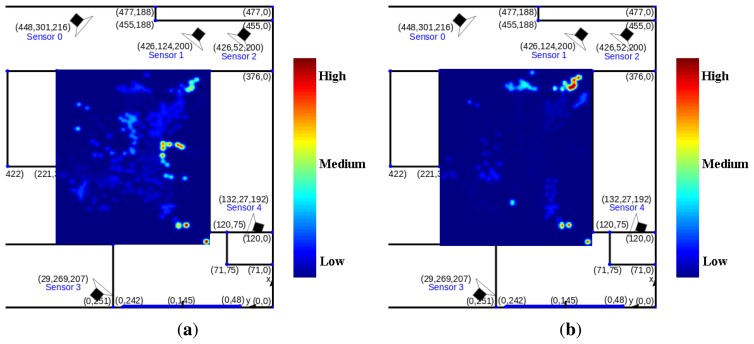
(**a**) Error map between average speed map of trajectories from our proposed tracker (RML) and UWB, and (**b**) Average TATE map of our proposed tracker.

The speed error map, which is the difference between speed maps RML and UWB, is also shown in Figure 13a. Speed error is higher in areas underneath the cameras and at the door, compared with other areas in the room. High speed error implies poor camera coverage in those areas. Finally, the heat map showing the TATE of RML for each discretized cell in the room is presented in Figure 13b. The largest errors occur near the door and near Sensor 1 and 2, where camera coverage is minimum.

Since the tracker of Berclaz *et al.*, requires frames from all five visual sensors (each sensors has two mouse sensors) at the fusion center, the data received by the fusion center is 30 × 30 × 10 = 9000 bytes (each frame captured by each mouse sensors is 30 × 30 pixels with image depth of 1 byte per pixel) and no feedback to the cameras. Each camera in our proposed tracker computes likelihoods at 49 hypothesis points around **ŝ***^m^*((*t* − 1)) and sends to the fusion center. Each log-likelihood value is represented with 16 bits (2 bytes) floating point number. Thus, the fusion center receives 49 × 2 × 10 = 980 bytes per person from 10 sensors. Thus, it is clear that our proposed tracker outperforms Berclaz *et al.*'s tracker in terms of inter-node communication efficiency for our test scenario.

Although sensors are equipped with digital signal controllers that are powerful enough to perform the video analysis (foreground/background segmentation, likelihood computation, *etc*.), our prototype tracker is implemented on a PC due to availability of rapid prototyping libraries such as OpenCV as well as an ease of parameter tuning and debugging. It is implemented in C++ and the videos are processed sequentially, *i.e.*, preprocessing, foreground detection and likelihood computation for video from each mouse sensors is done one after another before data fusion. The average processing speed on a single core Core™ 2 Quad CPU running at 2.66 GHz is about 200 fps. We are confident that the implementation of our tracker on digital signal controllers of visual sensors in distributed architecture (mentioned in Section 3) will be able to achieve real-time tracking at 33 fps.

## 6. Conclusions

In this paper, we propose a novel framework for automatically monitoring the mobility patterns of a person using a network of very low resolution sensors. The benefits of using low resolution sensors include, but are not limited to, lower cost, lower computational requirements and lower power consumption. The proposed mobility monitoring system allows tracking of a person of interest without relying on wearable sensors by exploiting privacy-preserving very low resolution images (30 × 30 pixels) provided by the visual sensor network. Based on statistical analysis of trajectories from the person trackers, the mobility patterns can be inferred. In comparison with other related works, we experimentally show that the accuracy of our tracker outperforms the state-of-the-art tracker by Berclaz *et al.*

Moreover, the mobility statistics, such as distance traveled and average speed, are extracted from each trajectory and compare with those extracted from ground truth UWB trajectories. The total distance traveled per sequence computed from trajectories of our proposed tracker shows that a person in Sequence 1 walks longer distance than a person in Sequence 2. Moreover, the average speed per sequence given by our tracker indicates that the person in Sequence 1 walks faster than the person in Sequence 2 on average. The statistics from ground truth UWB data makes the same conclusions. This shows that although the trajectories produced by our tracker are not perfect, they are accurate enough to derive useful statistics for mobility analysis.

Our prototype tracker is able to process videos from 10 mouse sensors sequentially at 200 fps on average on a single core of Core™2 Quad processor running at 2.66 GHz. This suggests the possibility of real-time tracking at 33 fps when implemented on digital signal controller of visual sensors using the proposed decentralized architecture.

Currently, the reported results are based on video data captured in semi-realistic environment, *i.e.*, captured in office room environment with person performing just basic mobility activities such as walking, standing and sitting. As a future work, we are going to perform extensive evaluation of our system's performance on real-life video data over long period (e.g., 6 months) of persons living in their home. Once an extensive evaluation of our system on the aforementioned real-life video shows satisfactory performance, the system will be eventually ported to digital signal controllers.

## Figures and Tables

**Table 1.** Results for the RML and POM-KSP trackers. The table shows the TATE results for raw tracks and for tracks after simple smoothing using a mean filter and outlier removal using a median filter. The b/g column shows the number of bad and good tracks.

**Sequence**	**RML**				**POM-KSP**			
	
	**Raw**	**Mean**	**Median**	**b/g**	**Raw**	**Mean**	**Median**	**b/g**
1	48.1655	41.339	42.3091	8/30	72.688	63.0252	63.6631	18/20
2	44.4059	38.6945	40.3259	5/21	69.4522	61.8375	64.9139	11/15

**Table 2.** Percentage of frames with TE larger than different threshold values for RML and POM-KSP.

	**Sequence 1**		**Sequence 2**	
	
	**TE > 100 cm**	**TE > 150 cm**	**TE > 100 cm**	**TE > 150 cm**
RML	1.2%	0.2%	6.5%	0.4%
POM-KSP	15.3%	7.5%	23.1%	13.9%
